# Phytochemical Profile, Preliminary Toxicity, and Antioxidant Capacity of the Essential Oils of *Myrciaria floribunda* (H. West ex Willd.) O. Berg. and *Myrcia sylvatica* (G. Mey) DC. (Myrtaceae)

**DOI:** 10.3390/antiox11102076

**Published:** 2022-10-21

**Authors:** Ângelo Antônio Barbosa de Moraes, Oberdan Oliveira Ferreira, Leonardo Souza da Costa, Lorena Queiroz Almeida, Everton Luiz Pompeu Varela, Márcia Moraes Cascaes, Celeste de Jesus Pereira Franco, Sandro Percário, Lidiane Diniz do Nascimento, Mozaniel Santana de Oliveira, Eloisa Helena de Aguiar Andrade

**Affiliations:** 1 Faculdade de Engenharia Química, Instituto de Tecnologia, Universidade Federal do Pará, Rua Augusto Corrêa, 01, Guamá, Belém 66075-900, Brazil; 2 Laboratório Adolpho Ducke, Coordenação de Botânica, Museu Paraense Emílio Goeldi, Av. Perimetral, 1901, Terra Firme, Belém 66077-830, Brazil; 3 Programa de Pós-Graduação em Biodiversidade e Biotecnología—Rede Bionorte, Universidade Federal do Pará, Rua Augusto Corrêa, 01, Guamá, Belém 66075-900, Brazil; 4 Laboratório de Pesquisas em Estresse Oxidativo, Universidade Federal do Pará, Rua Augusto Corrêa 01, Guamá, Belém 66075-900, Brazil; 5 Programa de Pós-graduação em Química, Universidade Federal do Pará, Rua Augusto Corrêa, 01, Guamá, Belém 66075-900, Brazil

**Keywords:** Amazonian natural products, *Artemia salina*, bioactive compounds, 1,8-cineole, (*Z*)-α-trans-bergamotene

## Abstract

The essential oils (EOs) of *Myrciaria floribunda* (Mflo) and *Myrcia sylvatica* (Msyl) (Myrtaceae) were obtained by hydrodistillation. The analysis of volatile constituents was performed by GC/MS. Preliminary toxicity was assessed on *Artemia salina* Leach. The antioxidant capacity was measured by the ABTS^•+^ and DPPH^•^ radical inhibitory activities. The results indicate that the Mflo EO had the highest yield (1.02%), and its chemical profile was characterized by high levels of hydrocarbon (65.83%) and oxygenated (25.74%) monoterpenes, especially 1,8-cineole (23.30%), terpinolene (22.23%) and α-phellandrene (22.19%). Regarding the Msyl EO, only hydrocarbon (51.60%) and oxygenated (46.52%) sesquiterpenes were identified in the sample, with (*Z*)-α-trans-bergamotene (24.57%), α-sinensal (13.44%), and (*Z*)-α-bisabolene (8.33%) at higher levels. The EO of Mflo exhibited moderate toxicity against *A. salina* (LC_50_ = 82.96 ± 5.20 µg.mL^−1^), while the EO of Msyl was classified as highly toxic (LC_50_ = 2.74 ± 0.50 µg.mL^−1^). In addition, relative to Trolox, the EOs of Mflo and Msyl showed significant inhibitory effects (*p* < 0.0001) against the DPPH^•^ radical. This study contributes to the expansion of chemical and biological knowledge on the EOs of Myrtaceae species from the Amazon region.

## 1. Introduction

The Amazonian flora is widely studied from chemical and biological perspectives due to the existence of species used in traditional medicine for the treatment of various endemic diseases in an effort to relate the practical uses of these species with the chemical composition of their natural products [1,2]. The essential oils (EOs) of aromatic members of the Amazonian flora are extensively studied by the scientific community due to their high-value-added properties; these organisms have also aroused industrial and economic interest due to their strong prospects for wealth generation and development in the region [3,4]. Among the families of Amazonian species that produce EOs, the Myrtaceae family stands out as one of the most important of the Neotropics, with great medicinal interest [5,6].

The family Myrtaceae is represented in Brazil by 29 genera and 1193 species, of which 266 occur in the Amazon [7]. Myrtaceae species are also economically important because many of them are edible and are sources of natural products with pharmacological potential [8,9,10,11,12,13,14]. The species of the family Myrtaceae are also widely known for their antioxidant potential, especially species of the genera *Eugenia, Syzygium, Myrciaria*, and *Eucalyptus* [15,16,17,18,19,20,21,22]. The volatile compounds present in the EOs of species of this family can eliminate or inhibit the effects of free radicals of oxidizing substances, attenuating the effects of oxidative stress and reducing the possibility of occurrence of degenerative diseases, such as Alzheimer’s disease, Parkinson’s disease, cancer, diabetes, and sclerosis [23,24,25]. The use of natural products as antioxidant substances is being increasingly studied today because they are environmentally friendly, cause no damage to the environment, and ensure greater safety of human health [26,27].

To determine possible biological activities, it is necessary to perform preliminary toxicity tests to evaluate the possible risks to human health [28,29]. Thus, the bioassay against the microcrustacean *Artemia salina* Leach is used to determine the level of cytotoxicity of natural products [30,31]. This test also allows the investigation of highly toxic EOs for possible uses as inputs in the manufacture of pest repellents and related products, especially in the agroindustry [32,33].

*Myrciaria floribunda* (H. West ex Willd.) O. Berg is one of the species of Myrtaceae found in the Amazon and is popularly known as “camboim” [34,35]. Its fruit is consumed in the form of gelatin or used as flavoring in the distilled beverage industry [36]. *Myrcia sylvatica* (G. Mey) DC. is a shrub popularly known as “cumatê-Folha-miúda”, “myrtle”, or “broom” that is used in folk medicine for the treatment of dysentery and intestinal diseases; like another species of Myrtaceae known as “pedra-ume-caá”, it also has potential for use against diabetes [37,38,39]. Despite the use of these species by the traditional communities of the Amazon, there are still few studies on the chemical profile and biological and antioxidant potentials of the EOs of these two species. Thus, the present study aimed to evaluate the phytochemical profile, antioxidant potential, and preliminary toxicity of the EOs of *M. floribunda* and *M. sylvatica* species.

## 2. Materials and Methods

### 2.1. Collection and Processing of Botanical Material

Shoots of *M. floribunda* and *M. sylvatica* were collected at Campina do Guarujá in the city of Bujaru, Lower Tocantins microregion, in the state of Pará, Brazil (01°57′36″ South latitude and 48°11′51″ longitude), in July 2017, following conventional botanical procedures. After collection, the material was dried in a convection oven at 35 °C for 5 days, ground, homogenized, weighed, and subjected to hydrodistillation to obtain the EO. The exsiccates, botanical identification, and registration were incorporated into the Collection of Aromatic Plants of the João Murça Herbarium collection of the Emílio Goeldi Paraense Museum, Belém, Pará, with the following registration numbers: *M. floribunda* MG237492 and *M. sylvatica* MG237516.

### 2.2. Production and Yield of Essential Oils

The EOs were obtained by hydrodistillation using a modified Clevenger apparatus for 3 h. After the end of distillation, the EOs were centrifuged for 5 min at 3000 rpm, dehydrated with anhydrous sodium sulfate (Na_2_SO_4_), and again centrifuged under the same conditions. Then, they were stored in amber glass ampoules and kept in a refrigerated environment at a constant temperature of 5 °C. The percentage of water in the studied samples was determined using an infrared moisture analyzer. The EO yield was calculated using the relationship between mass, oil, and moisture, as established by Santos et al. [40].

### 2.3. Identification of Chemical Components by GC/MS

The chemical composition of the EOs was analyzed in the Adolpho Ducke laboratory of the Goeldi Museum (LAD/MPEG, Belém, Brazil) by gas chromatography coupled to mass spectrometry (GC/MS) in a SHIMADZU QP Plus-2010 system equipped with a DB-5MS silica capillary column (30 m × 0.25 mm; 0.25 m film thickness) under the following operating conditions: carrier gas: helium, linear velocity of 36.5 cm.s^−1^; injection mode: splitless (2 µL of oil in 0.5 mL of hexane); injector temperature: 250 °C, temperature program: 60–250 °C with a gradient of 3 °C.min^−1^; temperature of the ion source and other parts: 220 °C.

The quadrupole scan rate was 39 to 500 daltons.s^−1^. Ionization was performed in electron impact mode at 70 eV. The identification of volatile components was based on the linear retention index (RI) and the fragmentation patterns in the mass spectra by comparison with standard samples in the NIST-11 and FFNSC-2 databases and from the literature [41]. The RIs were obtained using the homologous series of *n*-alkanes (C_8_-C_40_) from Sigma–Aldrich (San Luis, AZ, USA).

### 2.4. Preliminary Toxicity Bioassay with Artemia Salina

The preliminary toxicity of the EOs was tested against larvae of the microcrustacean *A. salina*. The *A. salina* cysts were incubated at room temperature (27–30 °C) under artificial lighting in an aquarium with artificial salt water: 46 g of NaCl, 22 g of MgCl_2_.6H_2_O, 8 g of Na_2_SO_4_, 2.6 g of CaCl_2_.6H_2_O, and 1.4 g of KCl in 2.0 L of distilled water. The pH was adjusted to 8.0–9.0 using Na_2_CO_3_.

Twenty-four hours after hatching, EO solutions were prepared in triplicate at concentrations of 100, 50, 25, 10, 5, and 1 µg.mL^−1^ using brine water as a vehicle and 5% dimethyl sulfoxide (DMSO) as a diluent. Ten larvae of *A. salina* in the meta-nauplius stage were placed in each tube. After 24 h, the mortality rate of the larvae was quantified, and the mean lethal concentration (LC_50_) was calculated using the Probitos statistical method, according to the methodology adapted from Góes et al. [42].

### 2.5. Tests of the Antioxidant Capacity of Essential Oils

#### 2.5.1. ABTS Assay

The radical inhibition activity (AIR) of 2,2′-azino-bis (3-ethylbenzothiazoline-6-sulfonic acid) diammonium salt (ABTS^•+^) was analyzed according to the initial principles proposed by Miller et al. [42] with the reaction conditions modified by Re et al. [43]. The method is based on the ability of substances to eliminate the cationic ABTS^•+^ radical, a blue-green chromophore with maximum absorption at 734 nm, resulting in the formation of the stable, colorless ABTS product.

Initially, ABTS (7 mM.L^−1^; Sigma–Aldrich; A1888; São Paulo/SP, Brazil) and potassium persulfate (140 mM.L^−1^; K_2_O_8_S_2_; Sigma–Aldrich; 216224; São Paulo/SP, Brazil) were mixed and left in the dark for 16 h to form the ABTS^•+^ radical (2.45 mM.L^−1^). Then, the radical was diluted with phosphate-buffered saline until reaching an absorbance of 0.700 ± 0.020 in an 800XI spectrophotometer (Femto; São Paulo/SP, Brazil) at 734 nm. Then, 30 μL of sample or standard was added to the solution (in triplicate), and after 5 min, the final absorbance was read. In addition, Trolox^®^ (1 mM.L^−1^; Sigma–Aldrich; 23881-3; São Paulo/SP, Brazil) was used as a standard antioxidant. We calculated the inhibition activity according to the following equation:

*AIR* (%) = [(*A_control_* − *A_sample_*)/*A_control_* × 100] (1)

where *A_control_* represents the absorbance of the ABTS^•+^ radical (2.5 mM.L^−1^), and *A_sample_* represents the absorbance of the sample.

#### 2.5.2. DPPH Assay

The AIR of 2,2-diphenyl-1-picrylhydrazyl (DPPH^•^) was determined according to the method proposed by Blois [44] with modifications. This assay assesses the total antioxidant capacity of a substance to eliminate the radical DPPH^•^ (Sigma–Aldrich; D9132; São Paulo/SP, Brazil), a violet chromophore with absorption at 517 nm, resulting in the formation of the hydrogenated DPPH product, which is yellow or colorless.

First, DPPH^•^ solution (0.1 mM.L^−1^) was prepared from the reaction between DPPH (394.32 g.mol^−1^; Sigma–Aldrich; A1888; São Paulo/SP, Brazil) and ethyl alcohol (PA; C_2_H_6_O; Sigma–Aldrich; 216224; São Paulo/SP, Brazil). Then, the absorbance of the DPPH^•^ solution was read in an 800XI spectrophotometer (Femto; São Paulo/SP, Brazil) at 517 nm. Next, 50 µL of the sample or standard (Trolox; triplicate) were mixed in 950 µL of DPPH^•^ solution and placed in a water bath at 30 °C for 30 min. Trolox was also used as a standard antioxidant. We calculated the AIR as described in the ABTS assay. Please refer to Supplemental Material S1, for better understanding of antioxidant methods.

### 2.6. Statistical Analysis

For analysis of the preliminary toxicity and ABTS^•+^ and DPPH^•^ AIR of the EOs of *M. floribunda* and *M. sylvatica*, analysis of variance (ANOVA (Analysis of Variance),) was applied. Significant differences were compared between groups using Tukey’s post hoc analysis. In all tests, a significance level of 5% (*p* ≤ 0.05) was considered.

## 3. Results and Discussion

### 3.1. Analysis of the Yield and Chemical Composition of Essential Oils

The following Table 1 shows the results for the yield and chemical composition of the EOs of the two species of Myrtaceae. In total, 26 volatile constituents were identified for *M. floribunda* and 30 for *M. sylvatica*.

The EO of *M. floribunda* predominantly contained hydrocarbon monoterpenes (65.82%) and oxygenated monoterpenes (25.74%). Tietbhol et al. [45] identified high levels of hydrocarbon sesquiterpenes (53.50%), oxygenated monoterpenes (16.70%), and oxygenated sesquiterpenes (16.50%) in the aromatic profile of *M. floribunda*. These results indicate a difference in chemical composition between the current sample and the sample reported in the literature. Regarding the EO of *M. sylvatica*, only hydrocarbon sesquiterpenes (51.60%) and oxygenated sesquiterpenes (46.52%) were observed in the sample. Raposo et al. [46] also identified high levels of hydrocarbon (28.00–63.90%) and oxygenated (15.30–51.40%) sesquiterpenes in the EO of the species.

#### 3.1.1. Yield and Chemical Composition of the Essential Oil of *Myrciaria floribunda*

The yield of the EO of *M. floribunda* in the present study was 1.02%. According to Tietbohl et al. [45], the yield of the EO of fresh leaves of *M. floribunda* originating in Rio de Janeiro, Brazil, was 0.37%. Barbosa et al. [47] reported a yield of the EO of *M. floribunda* fruits of 0.60%. These comparisons indicate that the yield of the present sample is higher than that recorded in the literature. Regarding the phytochemical profile of the EO of *M. floribunda*, the oxygenated monoterpene 1,8-cineole, also known as eucalyptol, was the component with the highest content (23.0%), followed by the hydrocarbon monoterpenes terpinolene (22.23%), α-phellandrene (22.19%), *o*-cimene (7.04%), and γ-terpinene (5.87%).

Tietbohl et al. [45] evaluated the chemical composition of the EO of the fresh leaves of a specimen of *M. floribunda* from the Restinga de Jurubatiba National Park, Rio de Janeiro, Brazil. According to the authors, 1,8-cineole (10.40%), β-selinene (8.40%), and α-selinene (7.40%) were the volatile constituents with the highest contents. The 1,8-cineole content found in the present study was twice that recorded by those authors. In addition, α- and β-selinene were not present in the chemical composition of the sample analyzed in this study.

Tietbohl et al. [45] emphasized that the EO of *M. floribunda* significantly increased mortality and interrupted the metamorphosis of the parasite *Trypanosoma cruzi*, indicating that the secondary metabolites present in the chemical composition of this natural product are promising for use as bioinsecticides and for the environmentally appropriate control of vectors of Chagas disease. Tietbohl et al. [48] reported that the EO of another specimen found in Rio de Janeiro, Brazil, was characterized by high levels of monoterpenes (53.90%), among which 1,8-cineole was the major component (38.40%), with a concentration higher than that indicated in the present study.

Barbosa et al. [47] analyzed the chemical composition of the EO of *M. floribunda* fruits collected in the Brazilian state of Pernambuco. According to the authors, the hydrocarbon sesquiterpenes δ-cadinene (26.84%) and γ-cadinene (15.69%) were the major components of the sample. De Oliveira et al. [49] reported β-(*Z*)-*o*-cymene (50.80%), 1,8-cineole (3.14%), γ-terpinene (2.51%), and (*E*)-caryophyllene (1.16%) as the constituents with the highest contents in the EO of lyophilized isolates of fruit of *M. floribunda* from the Restinga de Maricá in Rio de Janeiro, Brazil.

These results indicate that the phytochemical profiles of the EOs of the fruits and leaves are different. According to Ferreira et al. [50], species from different geographic locations have different chemotypes [50]. This statement may explain the differences between the chemical composition of the present sample and those of samples reported in the literature.

The major constituent of the sample (1,8-cineole) has potential as an anti-inflammatory drug for the treatment of cancer [51]. In addition, the encapsulation of 1,8-cineole in nanofibers can prolong fungal activities against *Candida* species given that the isolated compound is not a strong fungal agent [52,53,54]. 1,8-Cineole also has potential as an antimicrobial, repellent, and anticancer agent and can be used in the treatment of respiratory diseases, including COVID-19 [55,56,57,58,59,60,61,62,63].

This compound also has several industrial applications, mainly in the production of pharmaceuticals and as a flavoring of foods and toothpastes [64,65,66,67]. Recent studies also point to the use of this compound as a biosolvent and anti-corrosion agent, as it is an ecological alternative to synthetic products [68,69,70].

Terpinolene has larvicidal, insecticidal, antifungal, antibacterial, antiproliferative, cytoprotective, antiviral, antimicrobial, and antibacterial activities, with activity against dangerous multidrug-resistant bacteria in the treatment of industrial wastewater [71,72,73,74].

The compound α-phellandrene also has biological activities described in the literature, including potential antifungal properties, and could potentially be used as an endosomal gel for the treatment of gout [75,76]. Regarding *o*-cymene, recent studies point to possible antiviral effects against COVID-19 due to its anti-inflammatory and anti-influenza activities and as an antifungal agent [77,78,79]. There are few reports of the properties of γ-terpinene in the literature. However, according to Reis et al. [80], oils containing this compound as a major component or at high levels have moderate antimicrobial activity against food pathogens.

#### 3.1.2. Yield and Chemical Composition of *Myrcia sylvatica* Essential Oil

The yield obtained for the EO of *M. sylvatica* was 0.22%. According to Raposo et al. [46] The EO content of the leaves of a sample from Santarém, Pará, Brazil, ranged from 0.90 to 1.60%, reaching the highest content in the month of July (Amazonian dry season) and the lowest content in the month of January (rainy season). In addition, Raposo et al. [46] analyzed the yield of the EO of the fruit of the species and found a value of 1.70%. Saccol et al. [81] found an analyzed yield of 1.10% for EO from the dry leaves of a specimen from Santarém, Pará, Brazil. Rosa et al. [82] found a yield of 0.50% for the EO of *M. sylvatica*. These results indicate that the EO content of the present sample is lower than the results in the literature.

The EO of *M. sylvatica* analyzed in the present study showed the oxygenated sesquiterpene (*Z*)-α-trans-bergamotene as the volatile component with the highest content (24.57%), followed by the oxygenated sesquiterpene α-sinensal (13.44%) and hydrocarbon sesquiterpenes (*Z*)-α-bisabolene (8.33%), α-*trans*-bisabolene (7.06%), and β-*trans*-bisabolene (5.07%). Raposo et al. [46] evaluated the phytochemical profile of the EO of the leaves of a specimen of *M. sylvatica* collected in Santarém, Pará, Brazil. According to the authors, the hydrocarbon sesquiterpenes β-selinene (6.2–10.5%), cadalene (4.7–8.2%), α-calacorene (1.5–6.0%), δ-cadinene (0.0–6.0%), and *trans*-calamenene (3.5–6.5%) and the oxygenated sesquiterpene 1-*epi*-cubenol (5.9–9.8%) and muskatone (2.7–6.2%) characterized the chemical profile of the sample.

Raposo et al. [46] also evaluated the chemical composition of the EO of the fruit of *M. sylvatica* in the fertile period and found δ-cadinene (11.3%), β-selinene (6.0%), and 1-*epi*-cubenol (5.1%) as the volatile constituents with the highest contents. Saccol et al. [81] studied the volatile composition of the EO of the aerial parts of *M. sylvatica* from Santarém, Pará, Brazil. According to the authors, the hydrocarbon sesquiterpenes *ar*-curcumene (8.09%), β-selinene (6.48%), cadalene (6.24%), α-calamenene (5.89%), and (*Z*)-calamenene (5.54%) and the oxygenated sesquiterpene 1-*epi*-cubenol (7.41%) were the major components of the sample. The authors also found that this EO attenuated molecular and biochemical effects and physiological changes in *Rhamdia quelen* under different stress events.

Saccol et al. [83] analyzed the chemical composition of the EO of the aerial parts of *M. sylvatica* collected in Santarém, Pará, Brazil. According to the authors, α-selinene (16.08%), calamenene (11.68%), and α-calacorene (11.47%) were the volatile constituents with the highest contents. The authors also noted that this EO attenuates stress induced in the freshwater fish *Sparus aurata*. Saccol et al. [84] identified β-selinene (9.96%), cadalene (9.36%), α-calacorene (9.17%), and (*Z*)-calamene (8.17%) as major compounds of the EO of a specimen from Santarém, Pará, Brazil. Rosa et al. [82] identified (*E*)-caryophyllene (45.88%) and 14-hydroxy-(*Z*)-caryophyllene (10.15%) as the main components of the EO of a sample from Carolina, Maranhão, Brazil. According to Cascaes et al. [85], *M. sylvatica* has great genetic variability, which is directly responsible for the different chemotypes presented by the species.

Regarding the properties and applications of the major components, EOs containing high levels of (*Z*)-α-trans-bergamotene may have antibacterial and antifungal activities [86,87]. This compound is also used in industry as a flavoring agent and can be found in the aromas of cereals and other derivatives [88]. α-Sinensal is responsible for the sapodilla aroma and is also used in the food, cosmetics, and perfumery industries as a flavoring because it has an intense orange aroma accompanied by distinct floral notes [89,90]. Yi et al. [91] observed that α-sinensal showed antimicrobial activity and inhibited Gram-negative *Staphylococcus aureus* and *Bacillus subtilis*.

Lancaster et al. [92] showed that (*Z*)-α-bisabolene is a potential candidate insecticide against *Murgantia histrionica* (harlequin bug) and *Phyllotreta striolata* (flea beetle). There are few reports in the literature about the biological properties of the α- and β-*trans*-bergamotene isomers. However, Moraes et al. [93] stated that the two isomers are defensive components of insects common in Brazil. The potential of the two compounds and other derivatives as insecticides was also explored by Cribb et al. [94] for management of the southern green stink bug (*Nezara viridula*).

### 3.2. Preliminary Toxicity

In the *A. salina* bioassay, no mortality was observed for the control, showing that the use of 5% DMSO as a diluent is feasible [95]. The average lethal concentration (LC_50_) values of the EOs were calculated by fitting a logarithmic curve to the number of dead individuals and extracting the equation in Probitos. The table below shows the mortality results and the LC_50_ concentrations, with their respective coefficients of determination (R^2^) as well as the preliminary cytotoxicity classification of the EOs (Table 2).

The *A. salina* bioassay is used to efficiently evaluate the cytotoxicity of natural products aquatic environments; it is simple and fast and has low requirements [97]. The test allows the screening of a large number of toxic substances because the microcrustacean larvae are quite sensitive to various chemical constituents, and in some cases, toxicity to *A. salina* coincides with toxicity to mammalian cells [98,99].

Previous studies show that results of preliminary toxicity bioassay with *A. salina* vary according to the type of sample studied (essential oil or extract) and its chemical composition. For the brine shrimp bioassay performed with the essential oils obtained from *Conobea scoparioides* fresh and dried leaves, it was verified a CL_50_ equal to 7.8 ± 0.3 µg.mL^–1^ and 7.5 ± 0.3 µg mL^–1^, respectively. According to Ramos et al. [96], an EO is classified as toxic when LC_50_ is below 80 µg.mL^−1^, moderately toxic when LC_50_ is between 80 and 250 µg.mL^−1^, and nontoxic or slightly toxic when LC_50_ is higher than 250 µg.mL^−1^.

#### 3.2.1. Toxicity of the Essential oil of *Myrciaria floribunda*

The EO of the dry leaves of *M. floribunda* had a mean LC_50_ of 82.96 ± 5.20 µg.mL^−1^. This result indicates that the EO of *M. floribunda* is moderately toxic according to the classification of Ramos et al. [96]. Barbosa et al. [47] reported that the EO of *M. floribunda* showed high inhibitory activity of the enzyme acetylcholinesterase, the main target of *A. salina* and the compound responsible for the activity of the microcrustacean.

According to Barbosa et al. [100], the EO of *M. floribunda* showed moderate cytotoxic potential in mammalian host cells. However, the chemical composition reported by those authors differed significantly from that of the present sample because it had high levels of sesquiterpenes. According to Tietbohl et al. [48], the EO of *M. floribunda*, composed mainly of 1,8-cineole (38.40%), showed acute toxicity against *Oncopeltus fasciatus* and *Dysdercus peruvianus*.

According to Caldas et al. [101] and Izham et al. [102], 1,8-cineole, the major component of the present sample, showed no indication of toxicity in tests performed with mice. Izham et al. [102] also highlighted that 1,8-cineole had a cytotoxic effect against breast cancer cells without harming the health of the mice subjected to the test. Elmhalli et al. [103] showed that the EOs of *Salvadora persica* and *Rosmarinus officinalis*, which contain 1,8-cineole as a major constituent, have moderate toxicity against nymphs of *Ixodes ricinus.*

Bhowal and Gopal [104] stated that 1,8-cineole has no reported negative effects in animal experiments and that the toxicity reported thus far in animal experiments appeared only after the application of high doses. According to Galan et al. [105], the amount of 1,8-cineole used commercially does not produce harmful effects to human health, and the compound has toxic effects only when administered at high doses.

Ribeiro et al. [106] reported that terpilonene showed low acute toxicity by residual contact against the mite *Tetranychus urticae* and was less efficient than other monoterpenes. However, Agus [107] stated that terpinolene is among the most toxic monoterpenes along with α-terpineol and linalool. According to Scherf et al. [108], terpinolene was toxic to *Drosophila melanogaster*, with an LC_50_ of 34.60 μL.L^−1^ at 12 h of exposure.

Scherer et al. [109] found that terpinolene and α-phellandrene showed no cytotoxic effect against L929 fibroblasts or RAW 267.7 macrophages. Martínez et al. [110] stated that α-phellandrene exhibited high toxicity against larvae of the insect *Tenebrio molitor*. Abdelgaleila and El-Sabrout [111] indicated that the EO of *Schinus molle*, consisting of 29.78% α-phellandrene, was toxic to *Culex pipiens* mosquito larvae. Other compounds present in lower concentrations in the EO of *M. floribunda* may have toxic effects, such as γ-terpinene and γ-cymene, which were likely responsible for the toxic effect of the EO of *Eucalyptus camaldulensis* against several cancer cells [112].

The biological activities of EOs may be related to the presence of certain constituents at a higher content and to the synergistic and/or antagonistic effects exerted by all substances present in the samples [113,114,115,116,117,118]. Considering that 1,8-cineole has low toxicity, and the other compounds present at high levels have moderate or high toxicity, the combined effects of the volatile constituents of the EO of *M. floribunda* may explain its moderate toxicity. In addition, monoterpenes have low toxicity when compared to sesquiterpenes and phenylpropanoids [119,120,121].

#### 3.2.2. Toxicity of the Essential Oil of *Myrcia sylvatica*

Regarding the EO of *M. sylvatica*, the LC_50_ was 2.74 µg.mL^−1^, indicating that the natural product showed very pronounced toxicity against *A. salina* and that the oil of the species is highly toxic. This EO consists only of sesquiterpenes, which generally have high toxicity [122,123]. Many sesquiterpenes have shown promising potential for use as raw materials or additives in bioinsecticides, natural repellents, and biopesticides due to their high toxicities [124,125,126].

Rosa et al. [82] indicated that the EO of a specimen of *M. sylvatica* from Carolina, Maranhão, Brazil, showed toxicity to *A. salina*, with an LC_50_ of 79.44 µg.mL^−1^. The authors emphasized that the EO is composed mainly of sesquiterpenes, primarily (*E*)-caryophyllene and 14-hydroxy-(*Z*)-caryophyllene. Toxic effects of other species of *Myrcia* with high levels of sesquiterpenes have been described in the literature. Scalvenzi et al. [127] reported that the EO of *M. splendens* showed cytotoxic activity against the human tumor cell lines MCF-7 (breast) and A549 (lung) and the nontumor cell line HaCaT (human keratinocytes). Melo et al. [128] stated that the EO of *M. lundiana* showed toxicity against the insect *Acromyrmex balzani*.

According to Alves et al. [129], the major constituent of the EO of *M. sylvatica* is present in high content in the EO of *Cordia verbenacea*, which inhibited 25.9% of cowpea weevils at a concentration of 0.40 µL.cm^−3^. Powers et al. [87] indicated that the EOs of *Santalum album* and *S. paniculatum*, containing (*Z*)-α-trans-bergamotene as one of their major components, showed toxicity against Hep-G2 tumor cells (liver cancer). Regarding α-sinensal, there are no reports in the literature regarding its toxicity.

Fernandes et al. [130] found that the EOs of the leaves and stems of *Eremanthus erythropappus*, which contain (*Z*)-α-bisabolene at high levels, showed marked toxicity in mice by the MTT assay. Mahdavi et al. [131] showed that the EO of the *Zingiber officinale* rhizomes showed substantial toxicity against the potato moth (*Phthorimaea operculella*). According to the authors, this EO contains (*Z*)-α-bisabolene as one of its major components.

According to Fernandez et al. [132] α-*trans*-bergamotene was the major component of the EO of the fruits of *Garcinia gardneriana* and showed toxicity against *Aedes aegyptus* mosquito larvae. Vinturelle et al. [133] reported that the EO of *Copaifera officinalis* contained α-*trans*-bergamotene as one of the compounds with the highest content and caused 84.60% mortality in engorged females of the tick *Rhipicephalus microplus.* The authors attributed this toxicity to the presence of sesquiterpenes, including α-*trans*-bergamotene, in the chemical profile of the EO. Matsuda et al. [134] reported that the compound β-*trans*-bergamotene has moderate toxicity against cancer cells and has not yet shown satisfactory results for possible use as a drug.

The possible toxic effects of the five major components of the EO of *M. sylvatica* and the synergistic effects of the other sesquiterpenes present in the sample may have contributed to the high toxicity of the EO against the microcrustacean *A. salina* [135]. Thus, further studies should be conducted to evaluate the possible harmful effects of this EO and its major components on mammalian cells and human health in addition to its possible use as an additive for the production of larvicides, pesticides, and insecticides.

### 3.3. Antioxidant Capacity of Essential Oils

The antioxidant capacity of the EOs of *M. floribunda* and *M. sylvatica* was determined from the ABTS^•+^ and DPPH^•^ AIR and by comparison with Trolox (1 mM/L), a water-soluble synthetic analog of vitamin E and a potent antioxidant.

**Figure 1:**
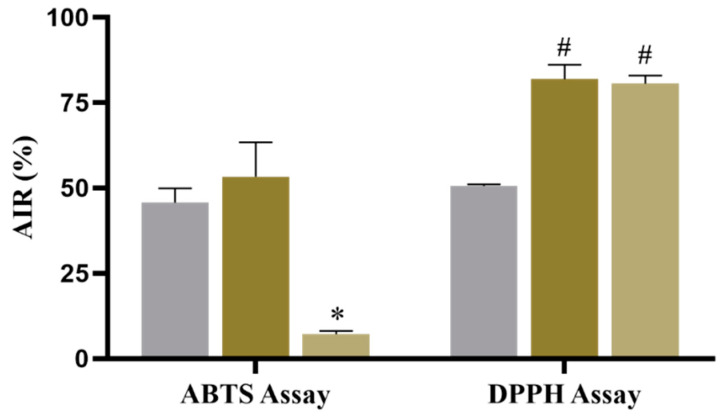
Percentage ABTS^•+^ and DPPH^•^ radical inhibition activity (AIR) of the essential oils of *Myrciaria floribunda* and *Myrcia sylvatica*. The results are expressed as the mean and standard deviation (*n* = 3). * *p =* 0.0001 versus Trolox and *Myrciaria floribunda*; ^#^ *p* = 0.0001 versus Trolox. 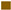 = *Myrciaria floribunda;* 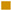 = *Myrcia sylvatica;* 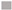 = Trolox.

According to Ferreira et al. [136], in the ABTS assay, the reaction between ABTS and K_2_O_8_S_2_ generates the ABTS^•+^ radical, which is then reduced again to ABTS in the presence of antioxidant compounds; the degree and time scale of this reaction are dependent on the capacity antioxidant concentration, concentration of antioxidants present in the Eos, and duration of the reaction between the compounds. According to the authors, in the DPPH assay, when the antioxidant power of the EO is high, the color of the solution changes from purple to yellowish over time due to the donation of a hydrogen atom or the transfer of electrons from substances in the EO to the DPPH^•^ radical, which then transforms into a stable diamagnetic molecule. In the present results, the RIAs of the EOs were proportional to their TEAC values (Figure 1).

#### 3.3.1. Antioxidant Capacity of the Essential Oil of *Myrciaria floribunda*

The results (Figure 1) showed that the EO of *M. floribunda* had an ABTS^•+^ AIR of 53.27 ± 8.27%, indicating that this EO had an antioxidant potential (*p* = 0.45) similar to that of Trolox (45.74 ± 4.16%). In the DPPH assay, the AIR of the EO was 81.91 ± 3.46%, indicating that the effect of the *M. floribunda* EO was superior to that of Trolox (*p* < 0.0001), with 50.53 ± 0.52%. These results indicate that the EO of *M. floribunda* has excellent antioxidant activity. This activity can be attributed to the chemical composition of the EO, mainly due to the presence of high levels of monoterpenes with antioxidant potential [137].

According to De Oliveira et al. [49], the EO of lyophilized fruits of *M. floribunda* showed high antioxidant potential toward the free radicals ABTS^•+^ and DPPH^•^. According to the authors, the antioxidant capacity of ABTS^•+^ free radicals was 550.14 μmol Trolox.g^−1^. In the DPPH^•^ assay, the EC_50_ was 85.68 g·g^−1^. Moreover, the EO was characterized mainly by hydrocarbon sesquiterpenes, and its 1,8-cineole content was almost eight times lower than that recorded in the present study.

There are few reported on the antioxidant potential of the EOs of other species of *Myrciaria*. However, Da Costa et al. [138] stated that *M. dubia* fruits are a natural source of antioxidants due to their substantial contents of ascorbic acid and phenolic compounds. In addition, the antioxidant potential of the components of *M. floribunda* and other species of *Myrciaria* are well-known, and further studies are needed to verify the antioxidant effects of their volatile components [139,140].

1,8-Cineole, the major component of the EOs in this study, is volatile and has been reported to have antioxidant potential [141]. EOs containing this oxygenated monoterpene as the major constituent have shown high capture power of the DPPH^•^ and ABTS^•+^ radicals, as demonstrated by Boukhatem et al. [142]. According to the authors, the EO of *Eucalyptus globulus* is composed of more than 94% 1,8-cineole and showed good results for the inhibition of DPPH^•^ radicals and a metal chelating activity superior to those of the synthetic antioxidants gallic acid and ascorbic acid. Limam et al. [143] also indicated that the EOs of *Eucalyptus* species with high levels of 1,8-cineole (>48.00%) exhibited a good ability to inhibit DPPH^•^ radicals.

Terpilonene is a constituent with important antioxidant activities highlighted in the literature [144]. Aydin, Turkez, and Taşdemir [145] stated that terpinolene showed excellent antioxidant capacity and great potential to inhibit oxidative stress. In addition, the authors showed that the compound’s antioxidant properties make it a strong candidate for anticancer treatment, but further studies are needed to corroborate these results. According to Osanloo, Ghanbariasad, and Taghinizhad [146], the EO of *Anethum graveolens* is composed mainly of α-phellandrene (26.75%). According to the authors, the natural product showed a good ability to capture DPPH^•^ radicals at concentrations above 160 µg.mL^−1^.

Other compounds at lower concentrations in the EO of *M. floribunda,* such as *o*-cymene, γ-terpinene, α-terpineol, myrcene, and α-pinene, also have antioxidant potential [147,148,149,150,151]. The synergistic and/or antagonistic effects of the constituents of this EO may explain its high capture of free radicals considering that the volatile components in the sample are known for their excellent antioxidant properties [152,153].

#### 3.3.2. Antioxidant Capacity of *Myrcia sylvatica* Essential Oil

According to the results (Figure 1), the EO of *M. sylvatica* exhibited an ABTS^•+^ AIR of 7.20 ± 0.72%, showing a lower effect than the EO of *M. floribunda* (*p* < 0, 0001) and the standard (*p* < 0.0001). On the other hand, in the DPPH assay, the AIR of the EO of *M. sylvatica* was 80.55 ± 2.00%; this effect was similar to that of *M. floribunda* oil (*p* = 0.99) and higher than that of Trolox (*p* < 0.0001). These data showed that the EO of *M. sylvatica* had different AIR effects. We believe that this difference in effects can be attributed mainly to the oxygenated sesquiterpenes present in the EO of *M. sylvatica*, which are highly reactive molecules due to their oxygenation content. In addition, the ABTS^•+^ radical exhibits steric hindrance around its nitrogen-centered atom, which hinders reactions with other highly reactive molecules, such as oxygenated sesquiterpenes.

Saccol et al. [84] evaluated the antioxidant effects of *M. sylvatica* EO on the sedation process of the tambaqui (*Colossoma macropomum*). According to the authors, the total reactive antioxidant potential in the brain and gills of anesthetized tambaqui was higher than that of the control. Franco et al. [154] analyzed the antioxidant capacity of the EO of *M. tomentosa*. According to the authors, the EO showed a high percent inhibition of free radicals DPPH^•^ (53.60 ± 0.15%) and ABTS^•+^ (213.00 ± 0.91). The authors also emphasized that this EO is characterized by high levels of hydrocarbon (56.74–75.82%) and oxygenated (15.09–16.83%) sesquiterpenes, mainly γ-elemene, germacrene D, (*E*)-caryophyllene, spathulenol, and α-zingiberene.

Gatto et al. [155] stated that the EO of *M. hatschbachii* had a low capacity to inhibit DPPH• free radicals (9.14 ± 0.33%). The authors also showed that the EO is composed mainly of hydrocarbon (47.81%) and oxygenated (30.05%) sesquiterpenes, mainly *trans*-calamenene (19.10%), (*E*)-caryophyllene (10.96%), and spathulenol (5.03%). Scalvenzi et al. stated that the EO of *M. splendens* showed an antioxidant potential six times higher than that of vitamin E for the inhibition of DPPH^•^ free radicals. According to the authors, the EO is characterized by the presence of *trans*-nerolidol (67.81%) and α-bisabolol (17.51%).

Regarding the antioxidant properties of the major component of the EO of *M. sylvatica,* there are few reports in the literature. However, Mohankumar et al. [156] stated that (*Z*)-α-trans-bergamotene at high concentrations in the EO of *Santalum album* may be responsible for the antioxidant and oxidative-stress-modulation activities of the EO, possibly by direct elimination of free radicals and activation of the antioxidant defense system in vitro and in vivo. M. Yi et al. [91] showed that in addition to (*Z*)-α-trans-bergamotene, α-sinensal was one of the main factors responsible for the ABTS^•+^ free radical capture ability of the EO of *Citrus reticulata*. Saroglou et al. [157] demonstrated that the high concentration of sesquiterpenes, including (*Z*)-α-bisabolene, in the EO of *Teucrium royleanum* was responsible for the DPPH^•^ RIA.

There are no reports on the possible antioxidant potentials of α-*trans*-bergamotene and β-*trans*-bergamotene in the literature. In general, sesquiterpenes have a lower antioxidant profile than monoterpenes [158,159]. In addition, oxygenated compounds have a greater capacity for free radical scavenging and reduced deleterious effects of lipid peroxidation, especially alcohols, phenols, and enols, due to the presence of hydroxyl groups [160]. These properties may explain the antioxidant behaviors of the EOs of *M. sylvatica* and *M. floribunda* against the ABTS^•+^ and DPPH^•^ radicals.

## 4. Conclusions

The present study indicated that the EO of *M. floribunda* is characterized by high levels of hydrocarbon and oxygenated monoterpenes, predominantly 1,8-cineole, terpinolene, and α-phellandrene. Regarding the EO of *M. sylvatica*, only hydrocarbon and oxygenated sesquiterpenes were identified in the sample, among which (*Z*)-α-trans-bergamotene, α-sinensal, and (*Z*)-α-bisabolene were the volatile constituents with the highest contents. The preliminary toxicity results indicated that the EO of *M. floribunda* exhibited moderate toxicity against *A. salina*, while the EO of *M. sylvatica* showed high toxicity. In addition, the EO of *M. floribunda* exhibited a greater capacity to inhibit the DPPH^•^ radical. This study contributes to the knowledge of the aromatic profile and antioxidant and biological properties of species of the family Myrtaceae from the Amazon region.

## Figures and Tables

**Table 1:** Chemical composition of the essential oils of the two species of Myrtaceae.

Species			*M. floribunda*	*M. sylvatica*
Yield			1.02%	0.22%
IR_L_	IR_C_	Chemical Component	Area (%)	Area (%)
932	935	α-pinene	3.30	
988	991	Myrcene	2.67	
1002	1008	α-phellandrene	22.19	
1014	1018	α-terpinene	1.53	
1026	1030	*o*-cymene	7.04	
1024	1033	Limonene	1.00	
1026	1036	1,8-cineole	23.30	
1054	1059	γ-terpinene	5.87	
1086	1091	terpinolene	22.23	
1095	1100	linalool	0.81	
1174	1178	terpinen-4-ol	1.63	
1186	1191	α-terpineol	2.45	
1374	1375	α-copaene	0.57	0.50
1389	1387	β-elemene		0.52
1411	1410	α-*cis*-bergamotene		0.41
1417	1419	(*E*)-caryophyllene	2.21	4.82
1430	1425	β-copaene		0.16
1434	1429	γ-elemene		2.89
1432	1431	α-*trans*-bergamotene		7.06
1440	1451	(*E*)-β-farnesene		1.79
1457	1456	β-santalene		2.44
1452	1456	α-humulene	0.16	
1484	1477	germacrene D		1.30
1480	1481	β-*trans*-bergamotene		5.07
1489	1488	β-selinene	0.17	
1496	1491	valencene	0.04	
1500	1493	bicyclogermacrene		3.34
1496	1497	viridiflorene	0.51	
1506	1498	(*Z*)-α-bisabolene		8.33
1500	1502	α-muurolene	0.15	
1505	1504	β-bisabolene		4.27
1514	1506	β-curcumene		0.56
1502	1509	*trans*-β-guaiene	0.11	
1514	1511	(*Z*)-γ-bisabolene		0.56
1521	1519	β-sesquiphellandrene		3.94
1522	1525	δ-cadinene	0.70	
1529	1527	(*E*)-γ-bisabolene		0.92
1537	1537	(*E*)-α-bisabolene		0.76
1559	1554	germacrene B		1.96
1561	1564	(*E*)-nerolidol	0.61	
1564	1573	davanone B	0.04	
1577	1574	spathulenol		1.46
1586	1581	thujopsan-2-α-ol		1.73
1592	1595	viridiflorol	0.19	
1595	1597	cubeban-11-ol	0.06	0.56
1640	1645	*epi*-α-muurolol		1.74
1652	1651	α-cadinol		1.25
1670	1666	*epi*-β-bisabolol		0.57
1674	1675	(*Z*)-α-santalol		0.39
1690	1694	(*Z*)-α-trans-bergamotene	0.11	24.57
1755	1748	α-sinensal		13.44
1806	1802	nootkatone		0.81
	Hydrocarbon monoterpenes		65.83	0.00
	Oxygenated monoterpenes		25.74	0.00
	Hydrocarbon sesquiterpenes		4.62	51.60
	Oxygenated sesquiterpenes		1.01	46.52
	TOTAL		99.65	98.12

RI_L_, retention index in the literature [41]; RI_C_, retention index calculated from a homologous series of *n*-alkanes (C_8_-C_40_) in a DB5-MS column. Relative area (%) calculated based on the peak areas.

**Table 2:** Preliminary cytotoxicity of essential oils.

Species	Concentration (µg.mL^−1^)	Mortality (%)	LC_50_ (µg.mL^−1^)	R^2^	Classification (Ramos et al. [96])
*M. floribunda*	100 50 25 10 5 1	56.67 50.00 33.33 0.00 0.00 0.00	82.96 ± 5.20 ^b^	0.76	Moderate toxicity
*M. sylvatica*	100 50 25 10 5 1	96.67 93.33 86.67 70.00 56.67 40.00	2.74 ± 0.50 ^a^	0.88	High toxicity

Values are expressed as the mean and standard deviation (*n* = 3) of the preliminary toxicity. Results with different lowercase letters (b or a) demonstrate that they are statistically different from each other.

## Data Availability

Not applicable.

## Supplementary Materials

The following supporting information can be downloaded at: https://www.mdpi.com/article/10.3390/antiox11102076/s1, Supplementary Material S1, Detailed preparation method of the potential antioxidant activity.
