# Supplementary material for: Phytochemical Profile, Preliminary Toxicity, and Antioxidant Capacity of the Essential Oils of Myrciaria floribunda (H. West ex Willd.) O. Berg. and Myrcia sylvatica (G. Mey) DC. (Myrtaceae)
Source: Antioxidants (Basel). 2022 Oct 21;11(10):2076. doi: 10.3390/antiox11102076 (PMC9658195; doi:10.3390/antiox11102076)
Supplement: Supplementary file 1 [file antioxidants-11-02076-s001.zip › antioxidants-1943647-supplementary-publish.pdf]

Phytochemical Profile, Preliminary Toxicity and Antioxidant Capacity of the Essential Oils of *Myrciaria floribunda* (H. West ex Willd.) O. Berg. and *Myrcia sylvatica* (G. Mey) DC. (Myrtaceae)

Ângelo Antônio Barbosa de Moraes [1,2], Oberdan Oliveira Ferreira [2,3], Leonardo Souza da Costa [1], Lorena Queiroz Almeida [1], Everton Luiz Pompeu Varela [3,4], Márcia Moraes Cascaes [5], Celeste de Jesus Pereira Franco [2], Sandro Percário [3,4], Lidiane Diniz do Nascimento [2], Mozaniel Santana de Oliveira [2,]* and Eloisa Helena de Aguiar Andrade [1,2,3,5]

[1] Faculdade de Engenharia Química, Instituto de Tecnologia, Universidade Federal do Pará, Rua Augusto Corrêa, 01, Guamá, Belém 66075-900, Brazil
[2] Laboratório Adolpho Ducke, Coordenação de Botânica, Museu Paraense Emílio Goeldi, Av. Perimetral, 1901, Terra Firme, Belém 66077-830, Brazil
[3] Programa de Pós-Graduação em Biodiversidade e Biotecnología—Rede Bionorte, Universidade Federal do Pará, Rua Augusto Corrêa, 01, Guamá, Belém 66075-900, Brazil
[4] Laboratório de Pesquisas em Estresse Oxidativo, Universidade Federal do Pará, Rua Augusto Corrêa 01, Guamá, Belém 66075-900, Brazil
[5] Programa de Pós-graduação em Química, Universidade Federal do Pará, Rua Augusto Corrêa, 01, Guamá, Belém 66075-900, Brazil
* Correspondence: mozanieloliveira@museu-goeldi.br

Abstract: The essential oils (EOs) of *Myrciaria floribunda* (Mflo) and *Myrcia sylvatica* (Msyl) (Myrtaceae) were obtained by hydrodistillation. The analysis of volatile constituents was performed by GC/MS. Preliminary toxicity was assessed on *Artemia salina* Leach. The antioxidant capacity was measured by the $ABTS^{\bullet+}$ and $DPPH^{\bullet}$ radical inhibitory activities. The results indicate that the Mflo EO had the highest yield (1.02%), and its chemical profile was characterized by high levels of hydrocarbon (65.83%) and oxygenated (25.74%) monoterpenes, especially 1,8-cineole (23.30%), terpinolene (22.23%) and α-phellandrene (22.19%). Regarding the Msyl EO, only hydrocarbon (51.60%) and oxygenated (46.52%) sesquiterpenes were identified in the sample, with (*Z*)-α-*trans*-bergamotene (24.57%), α-sinensal (13.44%) and (*Z*)-α-bisabolene (8.33%) at higher levels. The EO of Mflo exhibited moderate toxicity against *A. salina* (LC_{50} = 82.96 ± 5.20 μg.mL^{-1}), while the EO of Msyl was classified as highly toxic (LC_{50} = 2.74 ± 0.50 μg.mL^{-1}). In addition, relative to Trolox, the EOs of Mflo and Msyl showed significant inhibitory effects (p <0.0001) against the $DPPH^{\bullet}$ radical. This study contributes to the expansion of chemical and biological knowledge on the EOs of Myrtaceae species from the Amazon region.

Keywords: Amazonian natural products; *Artemia salina*; bioactive compounds; 1,8-cineole; (*Z*)-α-trans-bergamotene.

Supplementary Material S1

ABTS Assay

PREPARATION OF PBS pH 7.2 SOLUTION

Isotonic saline solution with pH 7.2 was used as a solvent in the preparation of the reagents. To prepare the phosphate-buffered saline solution (PBS), 1.48g of Na_2HPO_4 (dibasic sodium phosphate), 0.43 g of NaH_2PO_4 (monobasic sodium phosphate), and 7 g of NaCl (sodium chloride) were weighed. Then, they were dissolved in 1 L of distilled water and the pH of the solution was adjusted in the pH meter to 7.2.

PREPARATION OF THE ABTS$^{\bullet+}$ STOCK SOLUTION

Diammonium salt of 2,2'-azinobis(3-ethylbenzothiazoline-6-sulfonic acid) – ABTS. ABTS$^{\bullet+}$ stock solution was prepared 16 h prior to dosing. A solution of ABTS diammonium salt at 7 mM.L^{-1} was mixed with a solution of potassium persulfate ($K_2S_2O_8$) whose final concentration was 2.45 mM.L^{-1}.

(A) ABTS 7 mM.L^{-1}: 0.0768 g----20mL of PBS

(B) $K_2S_2O_8$ 140 mM.L^{-1}: 0.7560 g-----20mL of PBS

Note: 352 μL of the solution (A) was removed and discarded. After that, solution (A) was mixed with 352 μL of solution (B). Thus, 20 mL of ABTS$^{\bullet+}$ stock solution was prepared. The final concentration of (A) in this mixture was 2.45 mM.L^{-1}.

PREPARATION OF THE ABTS$^{\bullet+}$ WORKING SOLUTION

To prepare the working solution, ABTS$^{\bullet+}$ stock solution was mixed with 200 mL of PBS in an Erlenmeyer flask until the absorbance at 734 nm was 0.700 ± 0.02. In this step, 2200 μL of ABTS$^{\bullet+}$ stock solution was used, and added to PBS, then homogenized, and the spectrum was read.

- Calibrate spectrophotometer at 734 nm reading, zeroing with PBS.

PROCEDURE

Initially, 2970 μL of the ABTS$^{\bullet+}$ working solution was placed in the cuvette, followed by the first reading (T0). Then, 30 μL of the sample or standard was transferred to the cuvette containing the ABTS$^{\bullet+}$ working solution and, after 5 min, the second reading (T5) was performed. The reaction was measured in a spectrophotometer (Femto 800XI; São Paulo/SP) at 734 nm.

CALCULATION OF RESULTS

The total antioxidant activity (TAA) of each sample was calculated using the following formula:

$$TAA = \frac{(T_0 - T_5)}{T_0} \qquad Eq.(1)$$

Afterwards, the corrected value of the total antioxidant activity (TAAc) of each sample was calculated by subtracting the TAA value of the samples (essential oil) and the standard TAA value found for the Blank:

$$TAAc = (TAA - TAA_{Blank}) \qquad Eq.(2)$$

Subsequently, we calculated the inhibition activity according to the following equation:

$$AIR\ (\%) = [(Acontrol - Asample)/Acontrol\ x\ 100] \qquad Eq.(3)$$

where $A_{control}$ represents the absorbance of the ABTS$^{\bullet+}$ radical (2.5 mM.L^{-1}) and A_{sample} represents the absorbance of the sample.

DPPH Assay

PREPARATION OF THE WORKING SOLUTION OF THE DPPH•

Initially, 0.5 L of the DPPH• working solution was prepared. For this, 0.0197 g of DPPH (0.1 mM.L^{-1}) was weighed on an analytical balance and mixed with ethanol (0.5 L). Then, the initial absorbance reading of the DPPH• working solution (0.1 mM.L^{-1} in ethanol) was performed. Subsequently, 950 μL of DPPH solution and 50 μL of sample (essential oil) or standard were mixed and placed in a water bath at 37 °C for 30 min. After this period, the reading was performed in a spectrophotometer (Femto 800XI; São Paulo/SP) at 517 nm.

CALCULATION OF RESULTS

The absorbance values obtained, we calculated the inhibition activity according to the following equation:

$$AIR\ (\%) = [(Acontrol - Asample)/Acontrol\ x\ 100] \qquad Eq.(4)$$

where $A_{control}$ represents the absorbance of the DPPH• radical (0.1 mM.L^{-1}) and A_{sample} represents the absorbance of the sample.
